# Contribution of different classes of glutamate receptors in the corticostriatal polysynaptic responses from striatal direct and indirect projection neurons

**DOI:** 10.1186/1471-2202-14-60

**Published:** 2013-06-20

**Authors:** Bianca J Vizcarra-Chacón, Mario A Arias-García, Maria B Pérez-Ramírez, Edén Flores-Barrera, Dagoberto Tapia, Rene Drucker-Colin, José Bargas, Elvira Galarraga

**Affiliations:** 1 División de Neurociencias. Instituto de Fisiología Celular, Universidad Nacional Autónoma de México, México, DF, México

**Keywords:** Synaptic integration, Glutamate, NMDA, AMPA, KAINATE, Striatum, Corticostriatal inputs

## Abstract

**Background:**

Previous work showed differences in the polysynaptic activation of GABAergic synapses during corticostriatal suprathreshold responses in direct and indirect striatal projection neurons (dSPNs and iSPNs). Here, we now show differences and similarities in the polysynaptic activation of cortical glutamatergic synapses on the same responses. Corticostriatal contacts have been extensively studied. However, several questions remain unanswered, e.g.: what are the differences and similarities in the responses to glutamate in dSPNs and iSPNs? Does glutamatergic synaptic activation exhibits a distribution of latencies over time *in vitro*? That would be a strong suggestion of polysynaptic cortical convergence. What is the role of kainate receptors in corticostriatal transmission? Current-clamp recordings were used to answer these questions. One hypothesis was: if prolonged synaptic activation distributed along time was present, then it would be mainly generated from the cortex, and not from the striatum.

**Results:**

By isolating responses from AMPA-receptors out of the complex suprathreshold response of SPNs, it is shown that a single cortical stimulus induces early and late synaptic activation lasting hundreds of milliseconds. Prolonged responses depended on cortical stimulation because they could not be elicited using intrastriatal stimulation, even if GABAergic transmission was blocked. Thus, the results are not explained by differences in evoked inhibition. Moreover, inhibitory participation was larger after cortical than after intrastriatal stimulation. A strong activation of interneurons was obtained from the cortex, demonstrating that polysynaptic activation includes the striatum. Prolonged kainate (KA) receptor responses were also elicited from the cortex. Responses of dSPNs and iSPNs did not depend on the cortical area stimulated. In contrast to AMPA-receptors, responses from NMDA- and KA-receptors do not exhibit early and late responses, but generate slow responses that contribute to plateau depolarizations.

**Conclusions:**

As it has been established in previous physiological studies *in vivo*, synaptic invasion over different latencies, spanning hundreds of milliseconds after a single stimulus strongly indicates convergent polysynaptic activation. Interconnected cortical neurons converging on the same SPNs may explain prolonged corticostriatal responses. Glutamate receptors participation in these responses is described as well as differences and similarities between dSPNs and iSPNs.

## Background

Members of all three families of ligand-gated ionotropic receptors for glutamate, named after their selective agonists: α-amino-3-hydroxy-5-methyl-4-isoxazolepropionic acid or AMPA-receptors (GluR1-4), N-methyl D-aspartate or NMDA-receptors (NR1, 2A-D, 3A, B), and kainate or KA-receptors (GluK1-5), are present in the striatum [1-5], however, their distinct roles during corticostriatal synaptic activation have not been completely described, in particular, differences and similarities in the glutamatergic responses of striatal projection neurons from the direct and indirect pathways.

Striatal projection neurons (SPNs) receive monosynaptic glutamatergic inputs from diverse cortical areas (e.g. [6-12]). Monosynaptic contacts between pyramidal neurons and striatal neurons of the direct and indirect pathways (dSPNs and iSPNs), as well as with striatal interneurons, have been described based on the small variation in latency of the synaptic events [3,13-22]. In contrast, polysynaptic corticostriatal contacts have received much less attention. Nevertheless, prolonged and late corticostriatal synaptic responses following single cortical stimulus that may last hundreds of milliseconds while sustaining repetitive discharge have been described *in vivo* and *in vitro*[23-27]. Here, we ask whether prolonged synaptic responses are in part due to glutamatergic inputs *in vitro*, suggesting polysynaptic and convergent corticostriatal synaptic activation. It is common knowledge that a main evidence of polysynaptic circuitry converging onto the same postsynaptic neurons is the wide time distribution of latencies, denoting different arrival times for the synaptic inputs (e.g. [28]). Late synaptic latencies spanning an amount of time more prolonged than that explained by monosynaptic events give rise to complex temporal and spatial synaptic integration as demonstrated by electromyography (e.g. [29]), current-clamp (e.g. [23,30]) and voltage-clamp (e.g. [31,32]) recordings in different neurons and circuits.

Late and variable arrival times of glutamatergic synaptic inputs have been shown to be generated when stimulation of groups of interconnected excitatory neurons converge onto the same postsynaptic cells (e.g. [23,27,30]). This happens because most interconnected neurons are in the vicinity of the first stimulated neurons and thus, polysynaptic convergence is the result of activating a coherent set of these neurons [23]. Cortical stimulation, *in vivo* and *in vitro*, can initiate recurrent burst firing in both cortical and striatal neurons [23,33] as well as polysynaptic activation of cortical and striatal circuits [23,25,27,30]. In contrast, stimulation within the neostriatum is expected to activate sparsely extended cortical axons coming from distant cortical areas that would unlikely conform a connected circuit generating delayed activation times. Therefore, a main hypothesis of the present work is that a late prolonged activation of excitatory inputs would be elicited by a single cortical field stimulus but not by a neighboring single intrastriatal field stimulus of the same strength and within a few microns of distance. An additional hypothesis is that prolonged responses could be generated from any cortical area. Evidence for these hypotheses means that long-lasting synaptic responses may be derived from local cortical stimulus generating polysynaptic chains of neighboring connected neurons activated in sequence (e.g. [30]). This local polysynaptic activation of afferents should not be possible from the striatum, thus explaining the differences in the responses.

This work shows that the three families of glutamate receptors have different roles in the generation of corticostriatal and intrastriatal responses in dSPNs and iSPNs. In particular, the isolation of responses from AMPA-receptors showed the expected distribution of early and late latency inputs compatible with polysynaptic cortical convergence during corticostriatal responses. In contrast, intrastriatal stimulation was unable to elicit such prolonged responses with the same stimulus. An understanding of corticostriatal prolonged responses is important since they sustain recurrent burst discharge (up-states) [33,34] that increases the probability of synchronization and correlated firing among neurons [35,36]. These responses are a requirement for the generation of reverberant dynamics present in the striatal microcircuit [35,37].

## Results

### CNQX-sensitivity of prolonged glutamatergic corticostriatal responses

**Figure 1:**
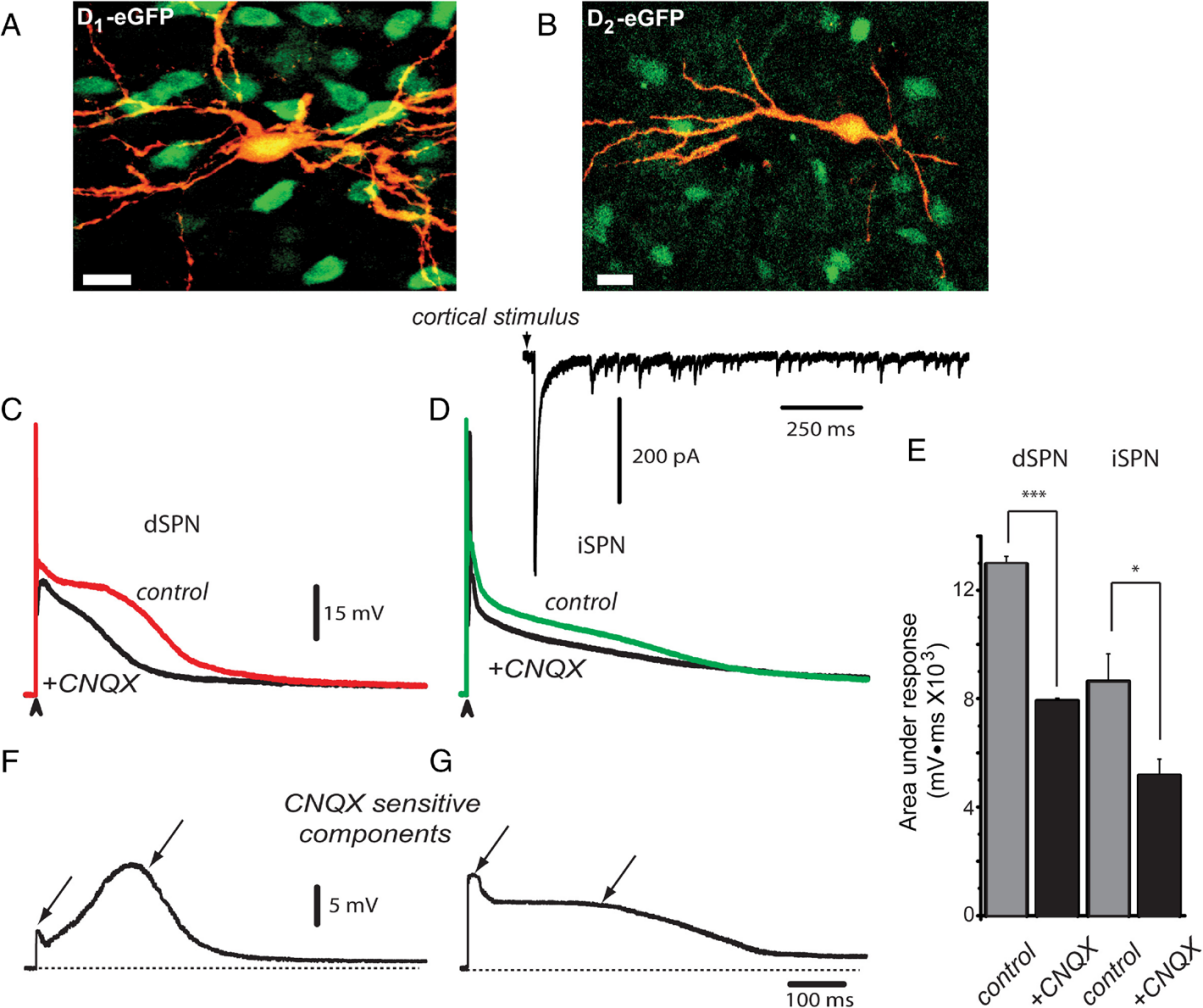
**Contribution of AMPA**/**KA**-**receptors in corticostriatal suprathreshold responses of SPNs. *A***: Photomicrographs of a double labeled recorded neuron. Superimposition of eGFP-green and injected biocytin-red-CY3 for a D_1_- eGFP. ***B***: Similar photomicrograph for a D_2_-eGFP neuron (scales = 10 μm). ***C***: Superimposed suprathreshold corticostriatal responses in a dSPN in control (red trace) and after adding 10 μM CNQX to the superfusion (black trace). ***D***: Superimposed suprathreshold corticostriatal responses in an iSPN in control (green trace) and after adding CNQX to the superfusion (black trace). The voltage-clamp recording in the inset shows that a cortical stimulus is followed by an initial PSC and a late barrage of PSCs that may last hundreds of milliseconds (stimulus strength was tuned up to reveal synaptic components only). ***E***: Histogram representing a sample of neurons shows that the CNQX-sensitive fraction of corticostriatal response in both dSPNs and iSPNs is about 40% in each case (***P < 0.001; *P < 0.05). ***F***: Digital subtraction of the CNQX-sensitive fraction from corticostriatal response in dSPNs shows two components (arrows): an early fast rising PSP followed by a late slow depolarization, suggesting polysynaptic arrival. ***G***: Digital subtraction of CNQX-sensitive fraction from corticostriatal response in iSPNs shows two components (arrows): an initial fast PSP followed by a late slow plateau, which underlies the corticostriatal response.

124 identified SPNs were recorded for the present study from PD30-60 BAC D_1_ or D_2_ eGFP mice and from rats of similar age (n = 70 dSPNs and n = 54 iSPNs). Figure 1A illustrates dSPNs neurons expressing eGFP and one neuron double labelled with biocytin (red CY3; see: Methods). Similar photomicrograph for a D_2_-eGFP neuron is shown in Figure 1B (scales = 10 μm). The red trace in Figure 1C is a control suprathreshold response, after a single cortical stimulus, in a dSPN. The superimposed black trace is the response obtained, with the same stimulus, after addition of 10 μM CNQX (an AMPA/KA-receptors antagonist) to the bath saline. Superimposed green and black traces in Figure 1D illustrate the same experiment in an iSPN. Inset in Figure 1D illustrates a voltage-clamp recording after cortical stimulation: the initial PSC is followed by a barrage of PSCs of smaller amplitude; such a discharge may last hundreds of milliseconds. Subtractions of the CNQX-sensitive components can be seen in Figure 1F (dSPNs) and 1G (iSPNs). The duration of the CNQX-sensitive components (hundreds of milliseconds) preclude the possibility that they could be explained by monosynaptic events following a single stimulus. In addition, the responses exhibited latency partitions: a preceding early phase - corresponding to monosynaptic PSPs (Figure 1F, G left arrows) [3,14,15,18,21,22,26,38-42], followed by a late component (Figure 1F, G arrows). A late component denotes the continuous activation of cortical neurons because: first, it is evoked by stimulating within the cortex mostly in a parasagittal corticostriatal preparation, secondly, it is sensitive to CNQX (an antagonist of glutamatergic synapses), third, it is not monosynaptic but extends along time lasting hundreds of milliseconds after the stimulus is over [30-32]; a characteristic of polysinaptically derived responses. Because these prolonged responses are not caused by repetitive stimulation [35] but by a single stimulus, it is logical to infer that they may be caused by recurrent firing within a local microcircuit [23,33], which has been shown to be the manifestation of interconnected neurons within a neuronal ensemble [35]. Because in parasagittal slices the amount of thalamic fibers and terminals present in both the striatum and cortex would be an invariant variable it cannot be explained by current diffusion to the thalamus [12,14,22]. Because these responses cannot be evoked by intrastriatal stimulation with the same stimulus (see below), we infer they arise from local cortical ensembles [30].

**Figure 2:**
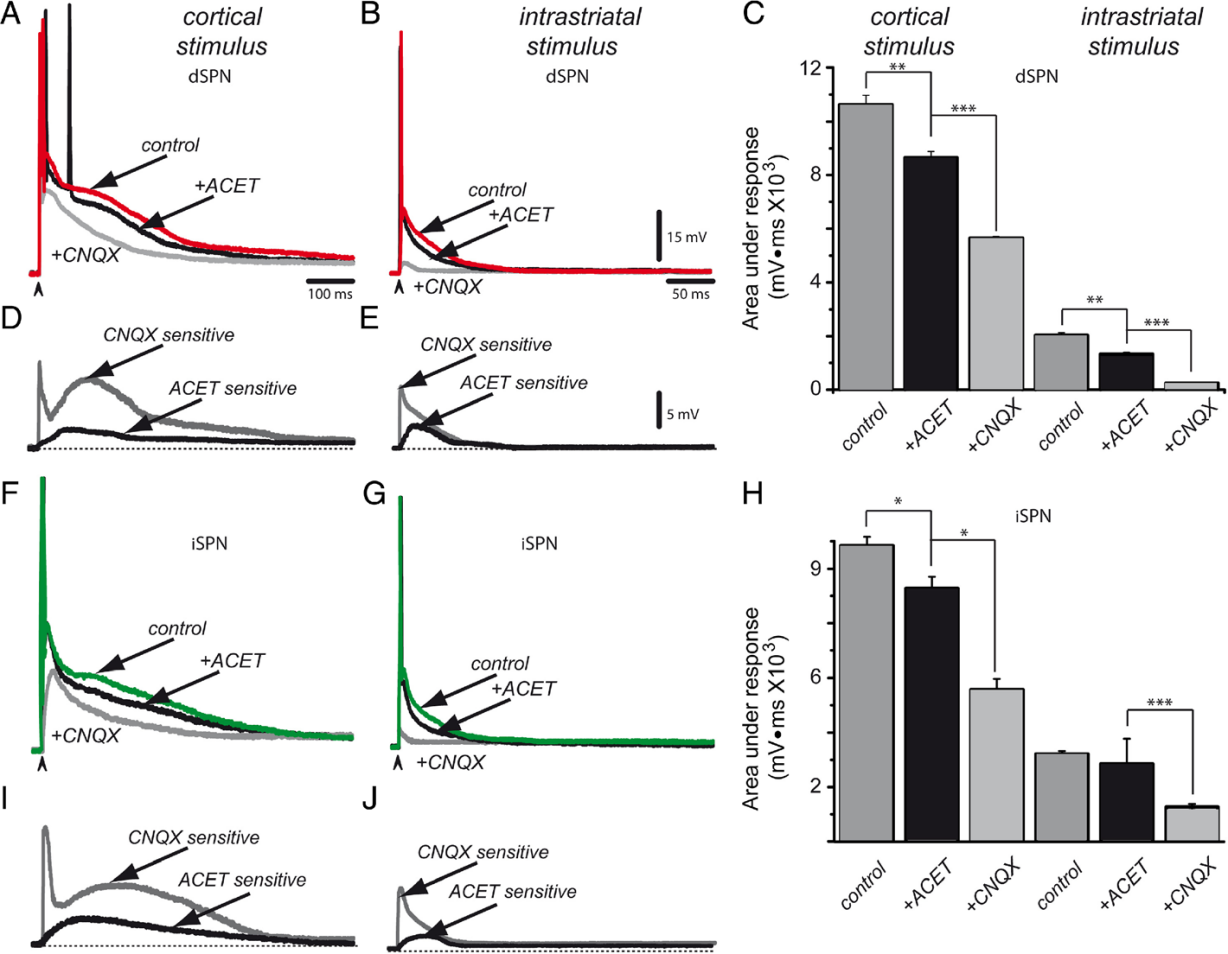
**Kainate-receptors can be activated by cortical afferents. *A***: Superimposed suprathreshold corticostriatal responses in a dSPN in control (red trace, control) and after adding 1 μM ACET to the superfusion (black trace, +ACET) to block KA-receptors while affecting, as less as possible, AMPA-receptors. After obtaining ACET-induced blockade, 10 μM CNQX were added to block remaining AMPA-receptors (pale grey trace, +CNQX). ***B***: Superimposed suprathreshold responses to intrastriatal stimulation in a dSPN in control (red trace) and after adding ACET (black trace). After obtaining ACET-induced blockade, 10 μM CNQX were added to block AMPA-receptors (pale grey trace). ***C***: Histogram summarizing similar results from a sample of experiments. ***D***: Digital subtraction of the ACET-sensitive component from corticostriatal response disclosed a slowly rising and decaying response, while supplemental CNQX-sensitive fraction exhibited both fast early and slow late responses in dSPNs. ***E***: Digital subtraction of ACET-sensitive constituent from response to intrastriatal stimulation mainly shows its contribution during PSP decay. Fast rising fraction is CNQX-sensitive. ***F***: Superimposed suprathreshold corticostriatal responses in an iSPN in control (green trace) and after adding ACET to the superfusion (black trace). Subsequent application of CNQX shows additional AMPA-receptors blockade (pale grey trace, +CNQX). ***G***: Superimposed suprathreshold responses to intrastriatal stimulation in an iSPN in control (green trace) and after adding ACET to the superfusion (black trace). Subsequent application of CNQX shows additional blockade (pale grey trace, +CNQX). ***H***: Histogram representing similar results from a sample of experiments). ***I***: Digital subtraction of ACET-sensitive component from corticostriatal response in iSPNs shows a slowly rising and decaying depolarization, while the CNQX-sensitive component shows fast early and late slow components. ***J***: ACET-sensitive component to intrastriatal stimulation in iSPNs mainly shows its participation during the decaying phase of the PSP. CNQX-sensitivity includes the fast rising PSP.

Area under corticostriatal responses in dSPNs decreased from 12,960 ± 288 mV · ms in control to 7,907 ± 103 mV · ms during CNQX for about 40% reduction (Figure 1E; n = 6; ***P < 0.001). In iSPNs CNQX reduced the response from 8,619 ± 1,033 mV · ms to 5,158 ± 613 mV · ms, again for about a 40% reduction (Figure 1E; n = 6; *P < 0.05). The similarities between dSPNs and iSPNs responses are that in both cases late responses last enough to be considered polysynaptic and contribute to about half the complex corticostriatal responses. A difference between dSPNs and iSPNs corticostriatal responses is that average areas under iSPNs responses are significantly smaller than those under dSPNs (P < 0.001; cf. Figure 2C and H) [25].

### Distinct synaptic contributions of KA- and AMPA-receptors during corticostriatal responses in SPNs

Post-synaptic KA-receptors are present in striatal neurons [2,39,43]. Here, we asked whether some class of KA-receptors could be synaptically activated by cortical afferents during suprathreshold corticostriatal responses (i.e.: with endogenous glutamate release). The physiological response of native KA-receptors may have different kinetics than AMPA-receptors [2,44,45] and we wanted to observe if this is true for corticostriatal responses. ACET (see Methods) has been reported as a highly selective antagonist for GluK1 KA receptor subunits in expression systems [46-48] and there are GluK1 subunits in the striatum [5,49] as reported by PCR techniques. GluK2, 3 and 5 subunits have also been detected [1,43,50,51]. KA-receptor heteromers made of different subunits have been reported to be blocked by ACET and other antagonists at low micromolar concentrations [52-54]. Splice and edited variants of these subunits associated with auxiliary proteins composing native receptors have unknown affinities for available antagonists [46,47,53]. Due to these considerations, we decided to try ACET to investigate its actions on corticostriatal physiological responses (0.1-1 μM ACET; see Methods) [47].

Red trace in Figure 2A shows a suprathreshold corticostriatal response in a dSPN. Superimposed black trace shows a reduction of control response during application of ACET. A subsequent application of 10 μM CNQX reveals an additional reduction due to blockade of remaining AMPA-receptors or KA-receptors insensitive to ACET (pale grey trace). Figure 2B shows a similar experiment during intrastriatal suprathreshold stimulation in the same dSPN. Histogram in Figure 2C shows that these actions were significant: blockade of KA-receptors in dSPNs decreased the area under the corticostriatal response from 10,633 ± 340 mV · ms to 8,649 ± 231 mV · ms for a 19% reduction (Figure 2C; n = 6; **P < 0.01). The subsequent blockade of AMPA-receptors reduced the remaining response to 5,640 ± 56 mV · ms for a supplemental 35% reduction (Figure 2C; n = 6; ***P < 0.001). ACET plus CNQX reduced the corticostriatal response by about one half: 45 ± 4% (P < 0.001), comparable to percent reduction obtained by CNQX alone (see above). Blockade of KA-receptors in dSPNs also decreased the area under the much briefer response obtained after intrastriatal stimulation (Figure 2B) from 2,031 ± 102 mV · ms to 1,337 ± 67 mV · ms for a 34% reduction (Figure 2C; n = 6; **P < 0.01). A subsequent application of CNQX reduced the remaining response to 241 ± 9 mV · ms or 82% (Figure 2C; n = 6; ***P < 0.001). Whole reduction by ACET plus CNQX blocked most intrastriatal response: ca. 90% (P < 0.001); implying different percent compositions of these components after corticostriatal (ca. 45%) or intrastriatal stimulation (ca. 90%).

Similar experiments were performed in iSPNs (Figure 2F-J), where green trace is the control and black trace shows the reduction produced by ACET. Subsequent addition of CNQX (pale grey trace) shows an additional reduction induced by blockade of remaining AMPA- or KA-receptors insensitive to ACET. Blockade of KA-receptors in iSPNs decreased the area under the corticostriatal synaptic response from 9,822 ± 330 mV · ms to 8,258 ± 430 mV · ms for a 16% reduction (Figure 2H; n = 6; *P < 0.05). A subsequent blockade of AMPA receptors reduced the remaining response to 4551 ± 397 mV · ms for a reduction of 45% (Figure 2H; n = 6; *P < 0.02). The combined action of both blockers in the original response amounted for about one half of the response: 46 ± 3% block (P < 0.01), not significantly different than the reduction obtained with CNQX alone (see above). ACET reduced the area under the response to intrastriatal stimulation from 2,203 ± 110 mV · ms to 1,842 ± 92 mV.ms for a 16% reduction (Figure 2H; n = 6; P < 0.1). Subsequent blockade of AMPA-receptors with CNQX reduced the remaining response to 254 ± 11 mV · ms for a larger block: 86 % reduction (Figure 2H; n = 6; ***P < 0.001).

Based on the above results we conclude that there are KA-receptors that respond to synaptic activation of cortical afferents (i.e.: endogenous glutamate release) in both dSPNs and iSPNs. Because the selectivity of ACET is still under debate (see above) we do not know whether the observed participation correspond to most or only some postsynaptic KA-receptors present in SPNs. Secondly, most glutamatergic actions during intrastriatal stimulation responses in dSPNs depend on AMPA/KA-receptors at −80 mV holding potential (Figure 2A, D, C). In contrast, response to ACET by iSPNs was more variable, although very clear in some cases (Figure 2G, J). But corticostriatal responses dissipate any doubt about ACET-sensitive responses in both dSPNs and iSPNs. However, in both neuronal classes only about half the corticostriatal response could be blocked by these antagonists. Therefore, cortical stimuli appear able to recruit a more complex response than intrastriatal stimuli, suggesting that the arrangement of cortical inputs is capable to activate more receptor classes [25,42,55]. In contrast, responses to intrastriatal stimulation go away almost completely after CNQX plus ACET, suggesting that recruited axons lack the necessary circuit arrangement for the response to build up.

In addition, subtractions of the KA- and CNQX-sensitive components (Figure 2D, E, I, J) disclose different time courses: the ACET-sensitive component is slowly rising and decaying and preferentially contributes during the late response. In contrast, the remaining CNQX-sensitive component participates in the fast early and the slow late response, suggesting that initial monosynaptic input is followed by a late polysynaptic barrage [30-32]. To conclude, a main difference between intrastriatal and corticostriatal responses is the duration and magnitude of the late response. More prolonged responses cannot be obtained stimulating within the striatum (even when GABA_A_-receptors are blocked, see below), suggesting a different arrangement or origin of afferents and synapses responding to the same stimulus at each site.

Because ACET may present some affinity problems (see above), we performed complementary experiments using the selective AMPA receptor antagonist, 25 μM GYKI 52466 (GYKI). The intention was to antagonize AMPA-receptors without affecting KA-receptors [1,48,56,57]. However, saturating concentrations of GYKI do affect KA-receptors [46,47,54]. Therefore, we used non-saturating concentrations of GYKI since our main aim was to find out whether AMPA-receptors are the ones responsible for the early and late latency components during corticostriatal responses.

**Figure 3:**
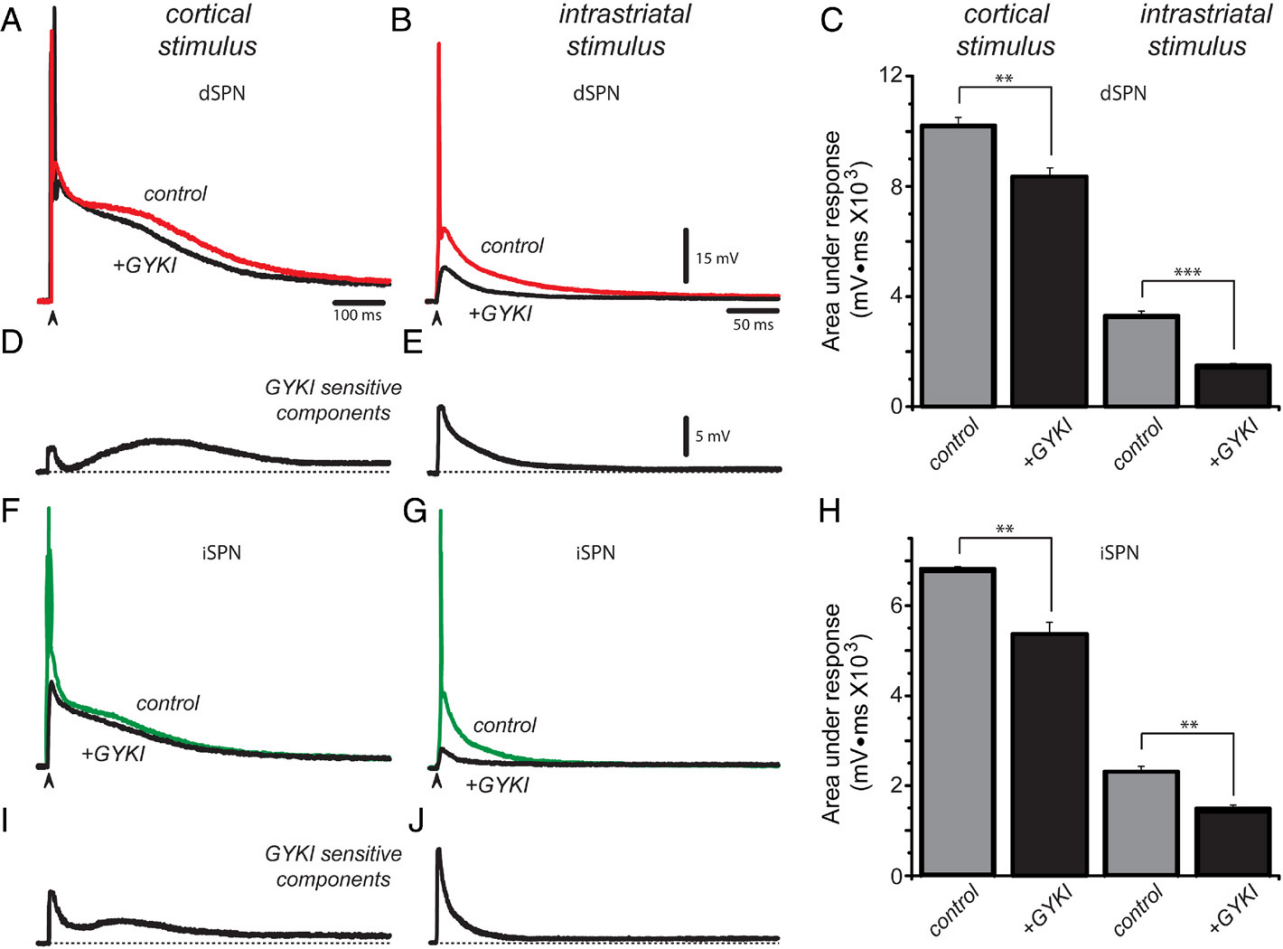
**Isolating AMPA-receptors contribution. *A***: Superimposed suprathreshold corticostriatal responses in a dSPN in control (red trace, control) and after adding 25 μM GYKI 52466 (GYKI) to the superfusion (black trace, +GYKI) to block AMPA-receptors while affecting, as less as possible, KA-receptors. ***B***: Superimposed suprathreshold responses to intrastriatal stimulation in a dSPN in control (red trace) and after adding GYKI to the superfusion (black trace). ***C***: Histogram summarizing results from a sample of neurons: the GYKI-sensitive fraction of corticostriatal response in dSPNs represents a 18% reduction (**P < 0.01), while GYKI-sensitive fraction of intrastriatal response in dSPNs represents a 55% reduction (***P < 0.001). ***D***: Digital subtraction of the GYKI-sensitive component from corticostriatal response shows both an early and a late response, indicating contributions to both mono- and poly-synaptic responses in dSPNs. ***E***: Digital subtraction of GYKI-sensitive component from response to intrastriatal stimulation mainly shows the initial response (previously reported AMPA-mediated PSP). ***F***: Superimposed suprathreshold corticostriatal responses in an iSPN in control (green trace) and after adding GYKI to the superfusion (black trace). ***G***: Superimposed suprathreshold responses to intrastriatal stimulation in an iSPN in control (green trace) and after adding GYKI to the superfusion (black trace). ***H***: Histogram representing results from a sample of similar experiments: GYKI-blockade represented about 21% reduction (**P < 0.01) for corticostriatal responses in iSPNs, while it represented about 36% reduction of responses to intrastriatal stimulation (**P < 0.01). ***I***: Digital subtraction of GYKI-sensitive component from corticostriatal response in iSPNs shows early and late components. ***J***: Digital subtraction of GYKI-sensitive component from intrastriatal response in iSPNs mainly shows the previously reported AMPA-mediated PSP.

Red traces in Figure 3A, B show control suprathreshold corticostriatal and intrastriatal responses, respectively, in a dSPN. Addition of GYKI (black traces in Figure 3A, B) reduced the responses. Histogram in Figure 3C shows that GYKI blockade was significant in both cases. Subtracted GYKI-sensitive components (Figure 3D and E; from experiments in Figure 3A and B, respectively) confirm that AMPA-receptors are responsible for early and late components of the response, suggesting that synaptic inputs arrive at different latencies; after the monosynaptic event that follows a single stimulus. As expected, the GYKI-sensitive fraction of the response to intrastriatal stimulation shows an almost complete absence of the late component with the same stimulus; as corresponding to a synaptic response evoked by a single stimulus.

Figure 3F and G show similar experiments in an iSPN, green traces denote controls and black traces denote recordings after 25 μM GYKI. Figure 3H shows that GYKI-induced reduction of the response was significant. As in dSPNs, subtracted GYKI-sensitive components show separate early and late components in the corticostriatal response, but only early responses after intrastriatal stimulus.

GYKI decreased the area under the corticostriatal response of dSPNs from 10,221 ± 292 mV · ms to 8,356 ± 313 mV · ms for an 18% reduction (Figure 3C; n = 7; **P < 0.01). In contrast, blockade of AMPA receptors in dSPNs during intrastriatal stimulation decreased the response from 3,314 ± 166 mV · ms to 1,500 ± 75 mV · ms for a 55% reduction (Figure 3B,C; n = 6; ***P< 0.001). GYKI decreased the area under the corticostriatal response of iSPNs from 6,815 ± 50 mV · ms to 5,374 ± 257 mV · ms for a 21% reduction (Figure 3H; n = 6; **P < 0.01). The action of GYKI on the responses to intrastriatal stimulation was from 2,306 ± 115 mV.ms to 1,486 ± 74 mV · ms (Figure 3H; n = 6; **P < 0.01) for about 36% reduction.

To conclude, the GYKI-sensitive fraction appears more important in the early latency component appearing after intrastriatal stimulation, while late latency components only appear clearly during corticostriatal responses. Taken together, the above data supports the idea that cortical stimulation activates cortical inputs in a way different than that employed during intrastriatal stimulus. One way to explain this difference has been posited *in vivo*[23]: cortical stimulus activates a group of interconnected excitatory neurons that converge onto the same postsynaptic SPNs. The arrival of these inputs at different latencies would prolong the responses. It is also observed that while AMPA/KA receptors almost completely explain the synaptic responses to intrastriatal stimulation, in the case of corticostriatal entries AMPA/KA receptors only explain about half the response. To explain this behavior one evidence has been given: polysynaptic activation not only involves cortical neurons [30] but also striatal GABAergic inputs (interneurons, other SPNs) eliciting a mixed excitatory plus inhibitory polysynaptic response [25]. Further experiments are needed to identify all neuronal classes participating. An additional component would be the contribution of NMDA-receptors.

### NMDA-receptor contribution in corticostriatal and intrastriatal responses of SPNs

**Figure 4:**
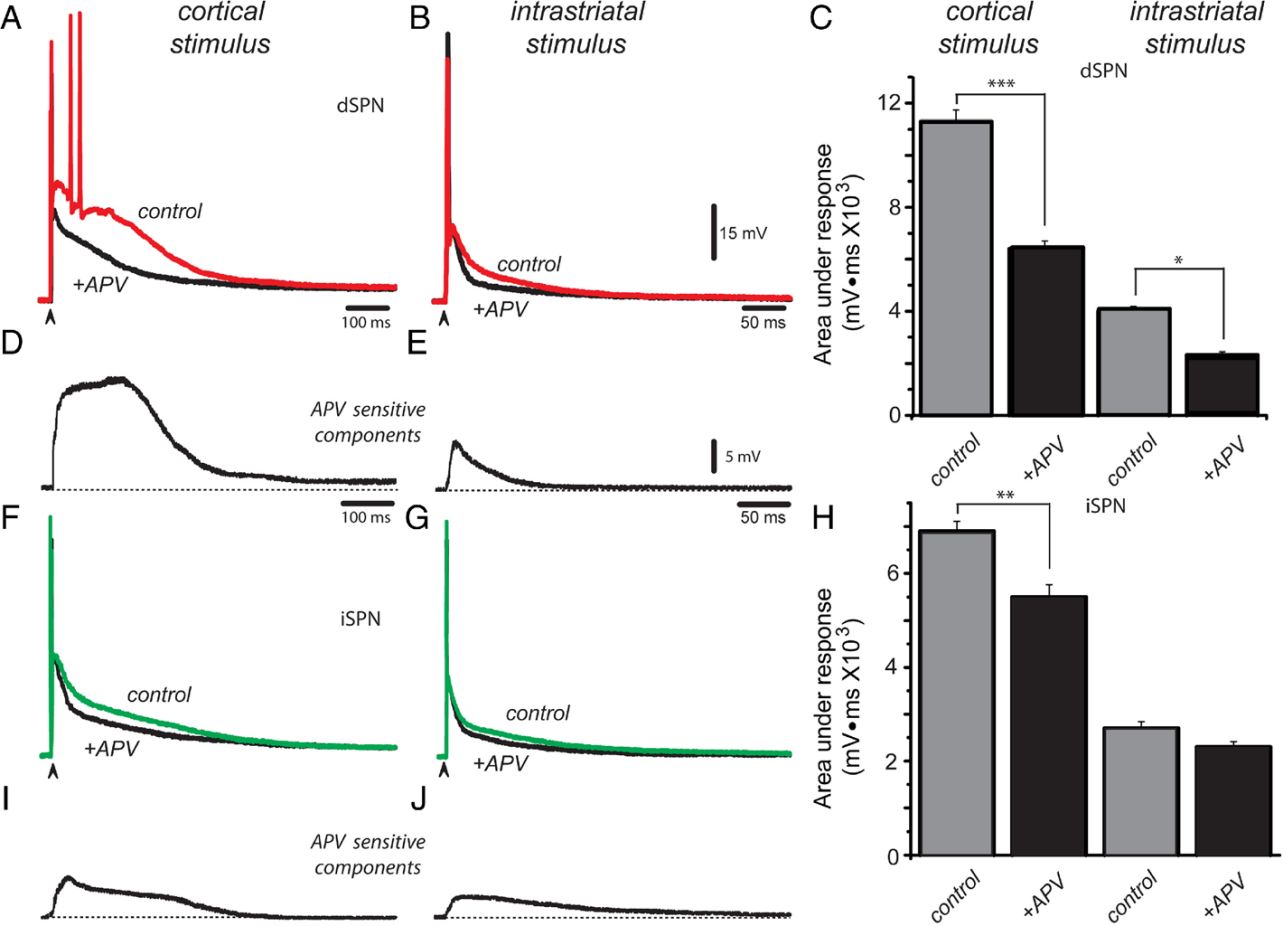
**Contribution of NMDA-receptors. *A***: Superimposed suprathreshold corticostriatal responses in a dSPN during control (red trace) and after adding 50 μM APV to the superfusion (black trace, +APV). ***B***: Superimposed suprathreshold response to intrastriatal stimulation in a dSPN in control (red trace) and after adding APV to the superfusion (black trace). ***C***: Histogram summarizing results in a sample of neurons: APV-sensitive fraction of corticostriatal response in dSPNs is 43% (***P < 0.001), while APV-sensitive fraction of intrastriatal response in dSPNs is 24% (*P < 0.05). ***D***: Digital subtraction of APV-sensitive component from corticostriatal response shows a quick rise and a plateau potential that most likely contributes to the duration of the response in dSPNs. ***E***: Digital subtraction of APV-sensitive component from intrastriatal response shows mainly the monosynaptic PSP previously reported by several authors. ***F***: Superimposed suprathreshold corticostriatal responses in an iSPN in control (green trace, control) and after adding APV to the superfusion (black trace, +APV). ***G***: Superimposed suprathreshold response to intrastriatal stimulation in an iSPN in control (green trace) and after adding APV to the superfusion (black trace). ***H***: Histogram representing results from a sample of neurons: APV-sensitive fraction of corticostriatal response in iSPNs is 21% (**P < 0.01), while APV-sensitive fraction of response to intrastriatal stimulation was statistically non-significant in the present sample (P > 0.05). ***I***: Digital subtraction of APV-sensitive component from corticostriatal response shows a smaller plateau potential than that found in dSPNs. ***J***: Digital subtraction of APV-sensitive component from response to intrastriatal stimulation shows a PSP slower than that shown in dSPNs.

Red trace in Figure 4A illustrates a control suprathreshold corticostriatal response obtained after a single stimulus in a dSPN and superimposed black trace shows the response obtained for the same stimulus after addition of an antagonist of NMDA-receptors, 50 μM APV, to the superfusion. APV decreased the magnitude of the response [14,15,18,33,35]. The same experiment is shown for suprathreshold responses to intrastriatal stimulation in the same neuron (Figure 4B). Histogram in Figure 4C shows that in both cases, APV-blockade was significant. Figures 4D and E show NMDA-sensitive responses (corresponding to experiments in Figure 4A and B, respectively). The APV-sensitive component was larger in corticostriatal responses. In contrast to the KA-sensitive component, the APV-sensitive component was fast rising. This can be explained by monosynaptic activation of NMDA-receptors [3,14,15,18,38,41]. In contrast to the GYKI-sensitive component (or the CNQX-sensitive component after ACET-blockade), the APV-sensitive response displays a persistent plateau depolarization instead of separate early and late components. Therefore, this response greatly explains the shape of the suprathreshold corticostriatal response in dSPNs and may be due to a number of factors: the slower kinetics of NMDA-responses, their capacity to produce plateau-potentials, and their capacity to activate intrinsic inward currents [15,18,33,55,58,59]. In contrast, the response to intrastriatal stimulation greatly resembles the gradual NMDA-mediated monosynaptic potential described in many neurons [58].

Figures 4F and G show a similar experiment in iSPNs. APV decreased corticostriatal and intrastrial dependent responses in various neurons, although decrease of responses to intrastriatal stimulation was more variable (Figure 4H). Interestingly, although subtracted APV-sensitive component in the corticostriatal response of iSPNs is of long duration (Figure 4I), the response is of lower amplitude (P < 0.001) than that of dSPNs and more similar to that found in other neurons [58]. Two phenomena have been reported to explain these differences: first, shorter and fewer dendrites in iSPNs make them more excitable [42], so that iSPNs are more prone to fire autoregenerative spikes during suprathreshold synaptic responses [25]. In turn, autoregenerative events trigger a stronger repolarization that reduces the amplitude of corticostriatal responses in iSPNs [58,60]. In support to this more integrative explanation, stimulation of dendritic spines locally with uncaged glutamate produces similar dendritic plateau potentials in both dSPNs and iSPNs [55].

In a sample of dSPNs, the area under suprathreshold cortical response goes from 11,256 ± 436 mV · ms to 6,395 ± 300 mV · ms after APV for a 43% reduction (Figure 4C, n = 9; ***P < 0.001). The decrease of intrastriatal responses was from 3,099 ± 155 mV · ms to 2,334 ± 117 mV · ms for a 24% reduction (Figure 4C; n = 6; *P < 0.05). The decrease in the area under the corticostriatal response in iSPNs after APV goes from 6,921 ± 206 mV · ms to 5,456 ± 266 mV · ms for a reduction of about 21% (Figure 4H; n = 6; **P < 0.01), while during responses to intrastriatal stimulation the reduction was from 2,722 ± 136 mV · ms to 2,280 ± 114 mV · ms for an average 16% decrease (Figure 4G,H; n = 6; P < 0.1), that is, variability and small amplitude of the response precluded statistical significance with this sample size, underlying a main difference with corticostriatal responses. Nonetheless, one example with a clear APV-sensitive component is illustrated in Figure 4J.

It was concluded that each cell class configures its response to glutamate differently, not by possessing different assortments of glutamate receptor classes in their synaptic contacts, but by the different use they make of the prolonged time window conferred by long-lasting synaptic activation: dSPNs apparently maintain larger APV-sensitive components (43%) than iSPNs (21%). This difference between dSPNs and iSPNs in part explains a smaller area under the response in iSPNs. In addition, prolonged time windows for synaptic integration can only be generated when stimuli are delivered in the cerebral cortex; where polysynaptic activation is virtually inescapable [30]. It cannot be generated when stimulus is delivered within the striatum: dSPNs (24%) and iSPNs (16%).

**Figure 5:**
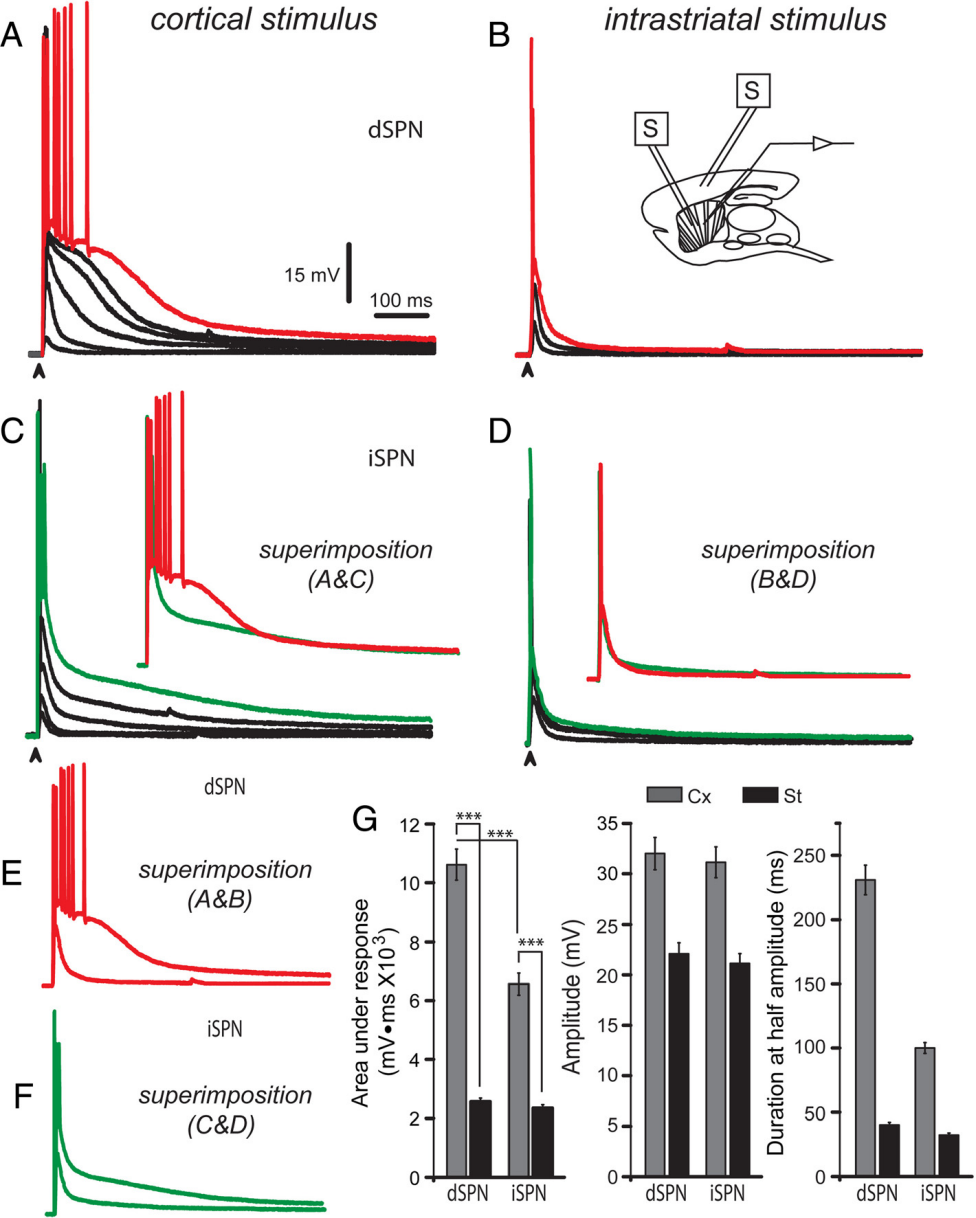
**Differences between dSPNs and iSPNs observed during corticostriatal synaptic integration are undetectable during intrastriatal stimulation. *A***: Corticostriatal responses evoked with increasing stimulus strengths until suprathreshold responses reach repetitive firing in a dSPN (red record). ***B***: Responses to intrastriatal stimulation with the same stimulus in the same dSPN. Inset: scheme illustrating positions of stimulation electrodes. ***C***: Corticostriatal responses evoked with increasing stimulus strengths until suprathreshold responses reach a brief spike burst in an iSPN (green record). Inset: a superimposition of corticostriatal responses in dSPNs and iSPNs. ***D***: Responses to intrastriatal stimulus evoked with the same stimulus. Inset: a superimposition of suprathreshold responses to intrastriatal stimulus in dSPN and iSPN neurons. ***E***: Superimposition of responses to cortical and intrastriatal stimulus in dSPNs. ***F***: Superimposition of responses to cortical and intrastriatal stimulus in iSPNs. **G**: Histograms comparing response areas, amplitudes, and duration at half amplitude (mean ± SEM). Note that only corticostriatal responses allow distinguish between dSPNs and iSPNs.

The last conclusion seems more dramatic when no receptor antagonists are used, repetitive discharge is allowed and traces are compared by superimposition (Figure 5): Comparison of suprathreshold corticostriatal responses shows clear differences between dSPNs and iSPNs (Figures 5A-D with insets) [25]. In contrast, superimposition of responses to intrastriatal stimulation cannot distinguish between dSPNs and iSPNs. Differences in the areas under the responses were: 10,612 ± 242 mV · ms (n = 19) for corticostriatal and 2,561 ± 128 mV · ms (Figure 5E, G; n = 12, ***P < 0.001) for intrastriatal stimulus in dSPN. In iSPNs, corticostriatal response area was: 6,527 ± 283 mV · ms (n = 16; P < 0.001); [25] and intrastriatal responses were 2,345 ± 117 mV · ms (Figure 5F, G; n = 10; ***P < 0.001). That is, there were significant differences in the corticostriatal responses between dSPNs and iSPNs (***P < 0.001) [25] but there were no significant differences in responses to intrastriatal stimulation between dSPNs and iSPNs. Therefore, antidromic activation of dispersed cortical axons within the neostriatum cannot activate interconnected converging cortical neurons, perhaps, because most interconnected neurons are in the vicinity of the ones stimulated first during a cortical stimulus [7,13,23]. Nevertheless, peak amplitude of responses had no significant differences between dSPNs and iSPNs, although, as reported previously [25], duration at half amplitude does show significant differences in corticostriatal responses between dSPNs and iSPNs. Here, we report that these differences are lost for intrastriatal responses (Figure 5G).

**Figure 6:**
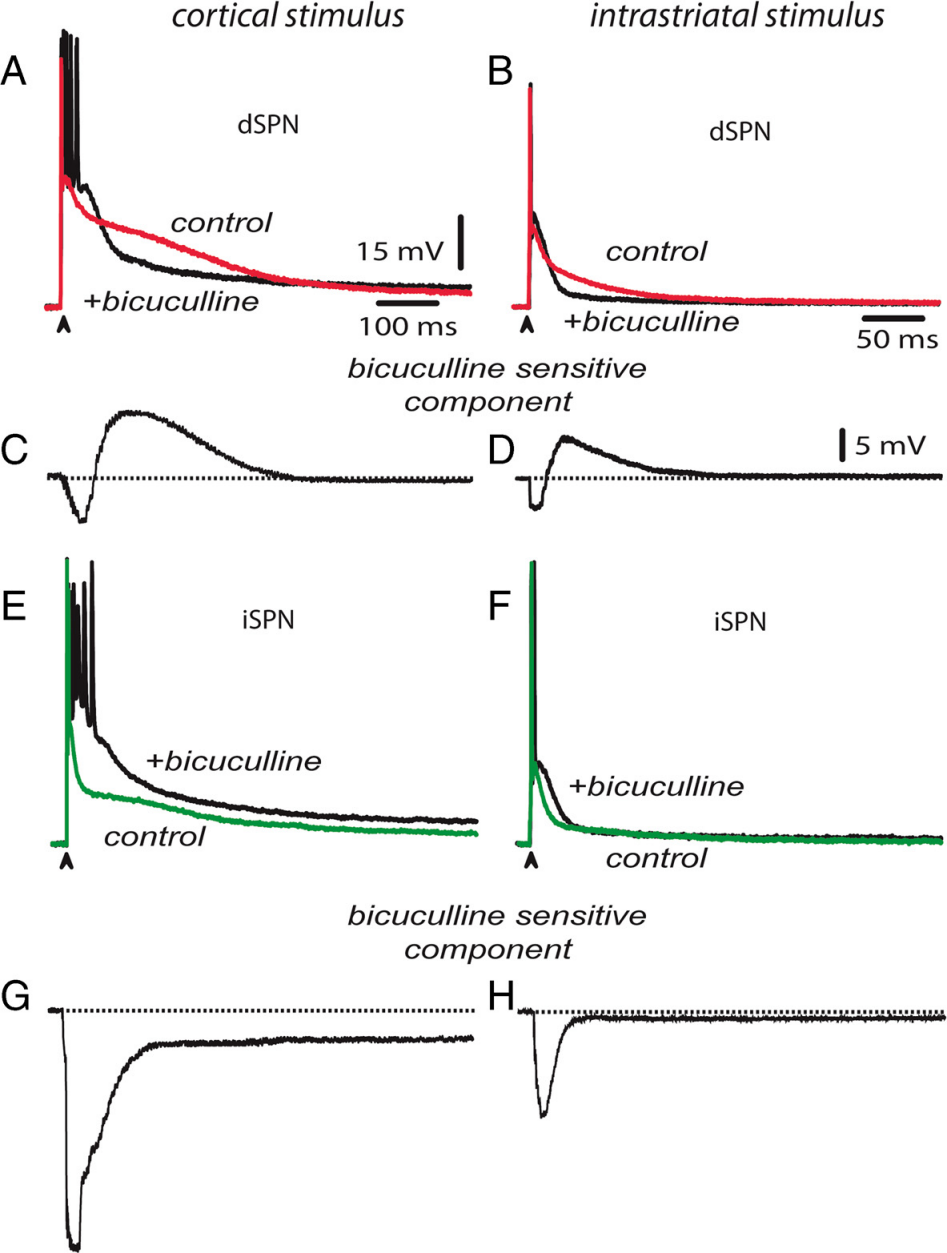
**More polysynaptic GABAergic inputs are evoked after cortical stimulation. *A***: A dSPN corticostriatal response before (red trace) and after the GABA_A_-receptors antagonist, 10 μM bicuculline (black trace), were added to the bath saline. ***B***: Similar experiment in the same dSPN but using intrastriatal stimulation. Bicuculline reduced both responses. **C**, ***D***: subtraction of responses in ***A*** and ***B***: bicuculline reduced the responses in both cases. Both responses exhibit hyperpolarizing and depolarizing phases (with respect to firing threshold). ***E***: A iSPN corticostriatal response before (green trace) and after 10 μM bicuculline (black trace). ***F***: Similar experiment in the same iSPN but using intrastriatal stimulation. Bicuculline enhanced both responses. ***G***, ***H***: subtraction of responses in ***E*** and ***F***: both responses exhibit hyperpolarizing actions (with respect to firing threshold).

Still, it can be argued that one reason for these differences in the responses depend on stimulation site: it may be that intrastriatal stimulation preferentially activates GABAergic inputs, which in turn, prevents the generation of prolonged synaptic responses. Experiments in Figure 6 show that this is not the case. Here, responses to corticostriatal stimulus are shown (Figure 6 left column) [25] to compare with responses to intrastriatal stimulus of the same strength, in the same cells (Figure 6 right column). Colored traces are controls and superimposed black traces are the responses to the same stimulus obtained after adding 10 μM bicuculline, a GABA_A_-receptor antagonist, to the bath saline. Blockade of GABA_A_-receptors not only did not prolong synaptic responses to intrastriatal stimulation in dSPNs, but instead, they reduced their duration even more. In dSPNs, a depolarizing GABAergic component contributes to the responses to both cortical and intrastriatal stimulation (Figure 6A, B) [25]. Subtracted bicuculline-sensitive responses show that activation of GABAergic inputs produce two different responses: they decrease the response of dSPNs at the beginning (Figure 6C, D) and enhance the response in a later phase; as it has been modeled [24]. Note that cortical stimuli are more efficient than intrastriatal stimuli to activate bicuculline-sensitive inputs onto dSPNs. In a sample of dSPNs, the area under the late latency component of the suprathreshold corticostriatal response was reduced from 8,268 ± 1014 mV · ms to 3,754 ± 1105 mV · ms after bicuculline for a 45% reduction (Figure 6A, C, n = 8; P < 0.025). The decrease of intrastriatal responses was from 1,874 ± 176 mV · ms to 960 ± 124 mV · ms for a 48% reduction (Figure 6B,D; n = 6; P < 0.001). On the contrary, areas under the corticostriatal responses in iSPNs after bicuculline were enhanced from 5,757 ± 623 to 9,560 ± 665 mV.ms for a 66% increase (Figure 6E, G; n = 11; P < 0.001), while during responses to intrastriatal stimulation the enhancement was from 1,988 ± 195 mV · ms to 3,035 ± 358 mV · ms for an average 52% increase (Figure 6F, H; n = 6; P < 0.002).

Two observations are evident: first, blockade of GABAergic inputs with bicuculline decreases the late depolarizing part of both corticostriatal and intrastriatal responses in dSPNs, while it enhanced both corticostriatal and intrastriatal responses in iSPNs [25]. Secondly, in both dSPNs and iSPNs activation of GABAergic polysynaptic inputs is larger for cortical than striatal stimulation [61] in absolute terms; although similar in percentage. In other words, dissimilarities in responses as a function of stimulation site could not be explained by differences in GABAergic activation.

**Figure 7:**
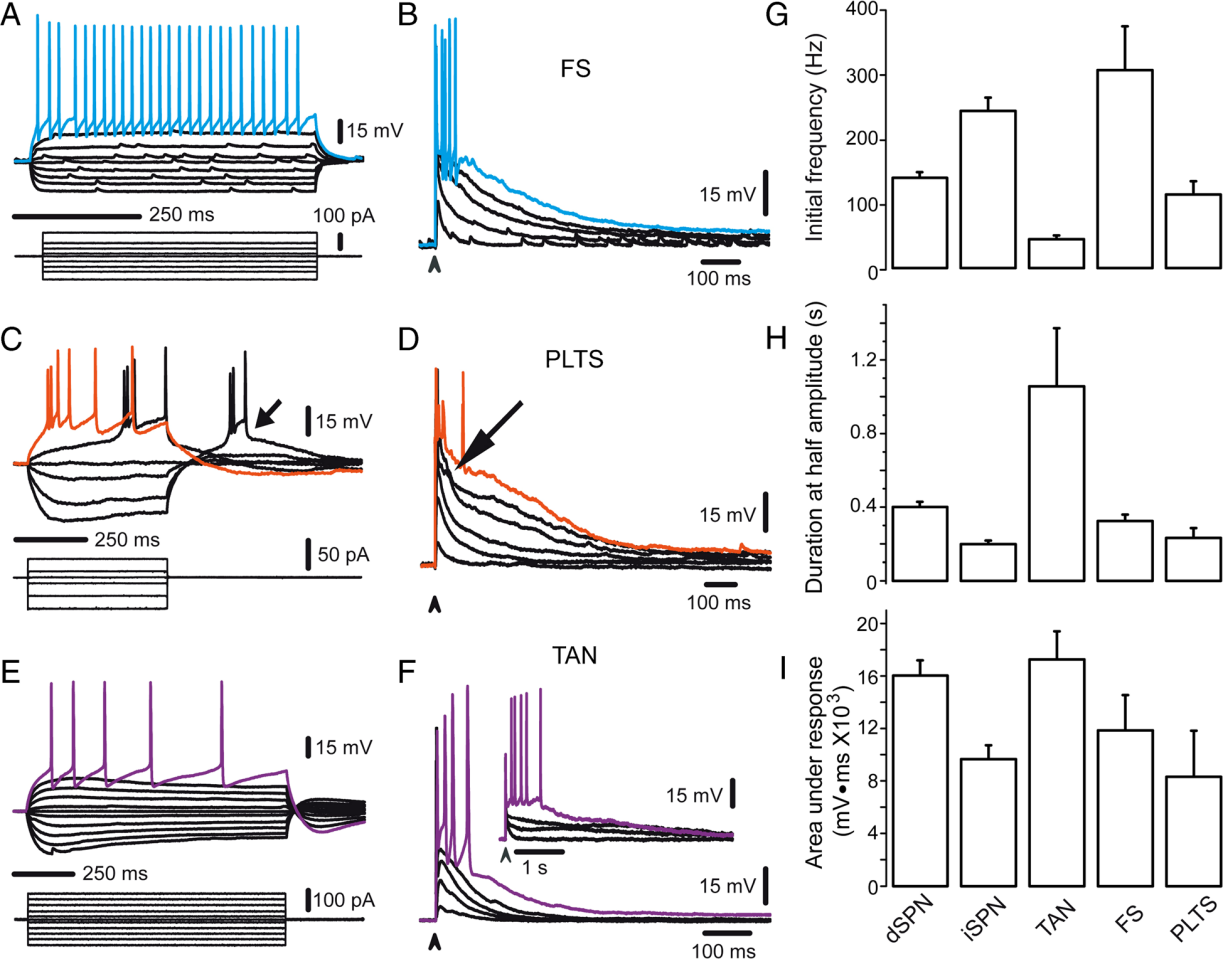
**Corticostriatal responses from striatal interneurons. *A***: Voltage responses (top) to intracellular current steps (bottom) from a fast-spiking (FS) interneuron. ***B***: Corticostriatal responses of the same FS interneuron to field stimulus of increasing strength. Suprathreshold responses (blue) elicit a high frequency discharge and last hundreds of milliseconds. ***C***: Voltage responses (top) to current steps (bottom) from a persistent low-threshold spike (PLTS) interneuron. Time scale was compressed as compared to A in order to observe the off-response (spikes are clipped). PLTS (arrow) elicits high frequency trains followed by slowly adapting action potentials. ***D***: Corticostriatal responses of the same PLTS interneuron. Holding potential (−80 mV) used for comparing responses is not the resting potential of these neurons so that action potentials on top of synaptic response appear partially inactivated. Still, synaptic depolarizations last hundreds of milliseconds after a single stimulus and some exhibit auto regenerative events (arrow). ***E***: Voltage responses (top) to current steps (bottom) from a tonically active neuron (TAN) at −80 mV holding potential. Note voltage sags after depolarizing and hyperpolarizing current steps. Evoked discharge is slowly adapting. ***F***: Corticostriatal responses of the same TAN interneuron evoked with field cortical stimuli. Synaptic responses last hundreds of milliseconds and even seconds (inset). Evoked discharge is of low frequency. ***G***: Histogram comparing frequency discharge (mean ± SEM in this and similar graphs) during corticostriatal responses in different interneurons: FS interneurons attain the highest frequencies while TAN interneurons attain the lowest ones. ***H***: Duration at half amplitudes is compared. Lengthier responses belong to TAN interneurons. ***I***: Areas under corticostriatal responses: largest areas correspond to TANs and dSPNs.

To further remark this point, experiments in Figure 7 show that main known classes of striatal interneurons are powerfully activated by a single stimulus from the cortex. The suprathreshold corticostriatal response of fast spiking (FS) interneurons (Figure 7A) [19,62-64] is shown in Figure 7B. It is a slowly decaying depolarization lasting hundreds of milliseconds with a high frequency spike train on top (Figure 7B, G): 305 ± 62 Hz (n = 7). In comparison, dSPNs discharge reaches 141 ± 7 Hz (Figure 7G; n = 10; P < 0.05), while the brief trains of iSPNs attain 248 ± 12 Hz under the same conditions (n = 7; NS). That is, during brief periods iSPNs may reach frequencies as high as those exhibited by FS interneurons. Mean latency for FS corticostriatal synaptic potentials (PSPs) was 1.56 ± 0.18 ms (n = 7; at 0.5X threshold; not shown), while the latency for similar responses in SPNs was 2.6 ± 0.22 ms (n = 46; P < 0.001), suggesting that both cortical and GABAergic inputs reach SPNs quasi-simultaneously [61], explaining why complex suprathreshold corticostriatal responses have mixed inhibitory and excitatory polysynaptic inputs thus comprising a feed-forward activating circuitry.

Interneurons that exhibit persistent low threshold spikes (PLTS) also exhibit prolonged corticostriatal depolarizations lasting hundreds of milliseconds after a single cortical stimulus (Figure 7C) [63,65,66]. However, they exhibit little output in terms of action potentials at these holding potential. Instead, they may exhibit autoregenerative events (“low threshold spikes”, arrows in Figure 7C, D), confirming that their way of activation may not necessarily arise from the polarized membrane potentials used for the present comparison (ca. -80 mV) [61]. Latency to subthreshold responses was 2.5 ± 0.3 ms (n = 6); not significantly different to that of SPNs. When action potentials are fired, initial frequency may reach 117 ± 17 Hz (Figure 7G).

Finally, tonically active neurons (TANs), known to be putative large aspiny cholinergic interneurons (Figure 7E) [65,67-70] may respond with repetitive firing after a single cortical stimulus, with maximal frequencies of 19 ± 6 Hz (n = 9), higher than those reached with intracellular current injections: 6–15 Hz (Figure 7E, F, G), but lower than those attained by any other striatal cell class. Nevertheless, corticostriatal latency to subthreshold PSPs in these neurons was 1.2 ± 0.11 ms, briefer than that of SPNs (P < 0.001), although not significantly different than that of FS interneurons. Depolarizing responses in TANs may, on occasion, last several seconds and be lengthier than those from any other striatal neuron (inset in Figure 7F, Figure 7H).

Taken together, the present results show: first, that GABAergic inputs from interneurons and other SPNs onto postsynaptic SPNs are more efficiently activated from the cortex than from the striatum itself, confirming that different GABAergic entries cannot explain differences in duration between corticostriatal responses and intrastriatal stimulus. Secondly, by themselves, interneurons responses to corticostriatal single stimulus are a direct proof that polysynaptic activation occurs during corticostriatal responses, not only involving cortical cells [23,30] but even striatal neurons [23,61], explaining the GABAergic component [25]. Finally, they show that not only corticostriatal responses between SPNs differ, but a comparison of areas under the responses as well as durations at half amplitude, as those seen in histograms of Figures 7G-I, is enough to confirm that each neostriatal neuron class exhibits a particular and distinct corticostriatal response, perhaps reflecting diverse combinations of cortical connections. This point has been shown for FS interneurons [13,19,62-64]. Further analysis of these differences is out of the scope of the present report.

### Corticostriatal responses do not depend on the cortical area being stimulated

**Figure 8:**
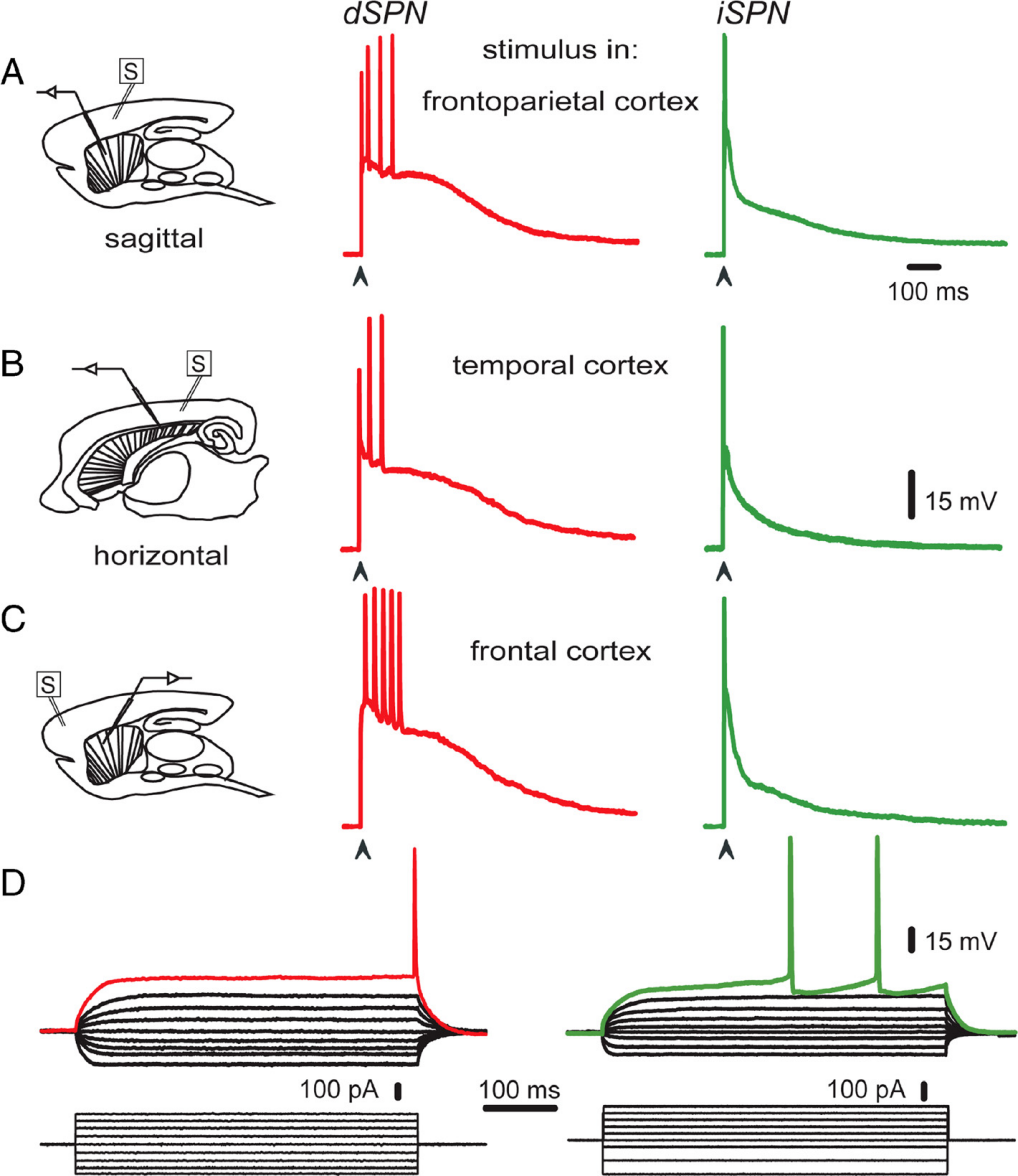
**Different corticostriatal synaptic responses of direct and indirect striatal projection neurons do not depend on the cortical area stimulated.** Corticostriatal suprathreshold synaptic responses were recorded after stimulus derived in the frontoparietal (***A***), temporal (***B***), and frontal (***C***) cortices (in sagittal or horizontal brain slices, schemes at left). It was observed that characteristic responses of dSPNs and iSPNs were maintained in any stimulated area, that is, the electrophysiological profile of each class was maintained. ***D***: Voltage responses (top) to intracellular current steps (bottom) recorded in dSPNs (left) and iSPNs (right).

Finally, it can be asked whether the corticostriatal responses that distinguish dSPNs from iSPNs are a result of stimulating a particular cortical location, or the use of a particular slice orientation. To answer these questions we stimulated different cortical areas with different slice orientations (Figure 8A-C): frontoparietal cortex (orientation = sagittal, n = 55 neurons, Figure 8A scheme at left), temporal cortex (orientation = horizontal, n = 15 neurons, Figure 8B) and frontal cortex (orientation = sagittal –shown–, and horizontal, n = 17 neurons, Figure 8C). It was observed that characteristic responses of dSPNs and iSPNs were maintained no matter the cortical area being stimulated [6-11] or whether the thalamus was present.

We conclude that differences in the responses were independent of the cortical area stimulated and on the assortment of receptors being activated, and more dependent on the different intrinsic properties that these neurons exhibit [60]. In fact, more differences have been documented after intrasomatic stimulation [42,60,71].

## Discussion

Here, we present evidence of two previously underestimated phenomena in *in vitro* corticostriatal slice preparations: First, that prolonged corticostriatal responses, evoked by stimulating cortical afferents and terminals with a single stimulus, are sensitive to ionotropic glutamate receptors antagonists, and therefore, may be the result of activating cortical neurons in sequence, in the vicinity of the stimulating electrode [30]. The activation of these cortical ensembles explains the continuous arrival of cortical inputs following a single stimulus, that is, they generate a polysynaptic activation that explains the prolonged late corticostriatal response. In support of this explanation, prolonged late components cannot be evoked by stimulating within the striatum (with or without the cortex or thalamus), but can be evoked by stimulating in any cortical area (with or without the thalamus). Secondly, this polysynaptic activation may in turn activate several striatal projection neurons and interneurons, thus explaining the GABAergic component of the complex corticostriatal response [25]. Third, KA-receptors contribute to these responses since they are activated by glutamate released endogenously by stimulated excitatory afferents. Fourth, the differences in the magnitude of the corticostriatal response between dSPNs and iSPNs is not due to different assortments of the main classes of glutamate ionotropic receptors, but by different integrative mechanisms that, among other things, produce larger APV-sensitive components in dSPNs.

### Prolonged corticostriatal responses

Previous evidences, *in vivo* and *in vitro*, have shown that a single cortical stimulus may initiate sequential polysynaptic activation of pyramidal cells [23,30].

Here, we show *in vitro*, that a single stimulus in the cortex evokes a complex and prolonged suprathreshold response, separate from the monosynaptic response, lasting hundreds of milliseconds, and sensitive to ionotropic glutamate receptors antagonists. Besides projection neurons (SPNs), several classes of striatal neurons including FS-, PLTS-, and TAN-interneurons could powerfully be activated by cortical stimuli, supporting and extending direct evidence of polysynaptic activation that includes not only cortical but also striatal neuron pools. It is important to demonstrate *in vitro*, that a long-lasting arrival of inputs may underlie prolonged corticostriatal responses: microcircuit dynamics demonstrated in the corticostriatal slice [35,36] is sustained by long-lasting up-states and recurrent burst firing [33] that allows neuronal synchronization. We infer that an up-state may be the result of the activity of a cortical ensemble. This information allows future comparisons and interpretations of changes in dynamics after decortication, or different forms of deafferentation, in control and Parkinsonian subjects.

Prolonged activation of excitatory inputs has been explained by the activation of neighboring cells by the first stimulated cortical neurons [12,23]. Sequentially activated cortical neurons [26] would then converge onto the same postsynaptic SPNs at different times, thus shaping up a response that may last hundreds of milliseconds setting a window for synaptic integration and correlated or synchronous firing [35]. Since striatal neurons are also activated by the stimulus, both cortical and striatal inputs together shape up the striatal output conveyed by SPNs.

An obvious question is why intrastriatal stimuli could not induce these prolonged responses. Our working hypothesis, confirmed so far, is that intracortical connections cannot be stimulated in these conditions, instead, stimulation within the neostriatum activates sparsely extended cortical axons coming from distant cortical areas that would unlikely be interconnected. Antidromic activation is not dense enough to stimulate neurons that are in the vicinity of the first stimulated cells [23]. The alternative explanation, that intrastriatal stimuli better activate inhibition, was discarded in the present work: it was shown that polysynaptic activation of inhibitory entries is larger when the stimulus is within the cortex and not within the striatum.

To conclude: accepting that cortical stimulation generates recurrent bursting and correlated firing due to the sequential activation of cortical neuron sets [30,33,72-75], and that these neurons sets may converge in target postsynaptic sets of SPNs, then, prolonged SPNs responses reflect circuitry processing among cortical and striatal neuron ensembles [34]. Striatal ensembles can also be activated in sequence [35], perhaps, encoding motor programs and procedures. Still an improbable alternative is that some thalamic inputs may be stimulated in a non equal manner by current diffusion. However, *in vivo* experiments point right toward the opposite phenomenon [76]. Finally, responses obtained *in vitro* are not equal to those evoked *in vivo*: a long-lasting disinhibition due to intracortical connections activating nets of inhibitory cortical interneurons [12,22,23,27] appears to be small or lacking in the present preparation.

### Roles of glutamate receptors during corticostriatal responses

Prolonged corticostriatal responses displayed GYKI-, ACET-, CNQX- and APV-sensitive components in both dSPNs and iSPNs. In particular, the contribution of GYKI- and CNQX-sensitive components in both neuron classes exhibited early and late components. We conclude that both projection neuron classes have qualitatively similar assortments of glutamate receptors. Therefore, differences between the magnitudes of orthodromically activated responses (e.g., areas under the response) are not due to differences in expressed glutamate receptors as experiments with uncaged glutamate confirm [55], but by different ways of integration due to anatomical and intrinsic differences (e.g., different sets of G-protein coupled receptors and modulation - unpublished). Taken all together, these results show that up-states appear not to be a simple spatial or temporal summation of otherwise uncoordinated synaptic inputs. They are the manifestation of ensembles processing and circuitry modulation.

The ACET-sensitive component suggests either that glutamatergic synapses in SPNs have GluK1 subunits [5,49], or that heteromers are sensitive to ACET [46,53]. The postsynaptic presence of an ACET-sensitive component with a physiological stimulus (release of endogenous glutamate) confirms previous suggestions about their potential postsynaptic importance [2,51].

A particular interest of the KA-receptors mediated responses is that their slow duration may ignite intracellular signaling cascades that cross-talk with other modulatory signaling cascades (e.g., dopaminergic; cholinergic). Further research is needed to observe this possibility as well as its interactions with voltage-gated currents.

## Conclusions

In previous works we showed the participation of GABAergic and intrinsic components in the complex and prolonged corticostriatal suprathreshold response [24,25,33]. In the present work, we demonstrate the participation of the three different classes of ionotropic glutamatergic receptors in the same response. Further, by isolating responses from GYKI- and CNQX-sensitive components out of the complex suprathreshold response of SPNs, a single cortical stimulus revealed that prolonged responses are due to the polysynaptic and sequential activation of cortical and striatal microcircuits converging on the same SPN; from either direct or indirect pathways. In fact, prolonged responses could not be evoked with intrastriatal stimulation, where apparently, the necessary arrangement of cortical connections to set the sequential and convergent activation cannot be activated with a single stimulus. Prolonged responses from APV- and ACET-sensitive components were shown to contribute to plateau depolarizations. However, the APV-sensitive component had lower amplitudes in iSPNs. GABAergic polysynaptic inputs were also shown to be recruited from the cortex.

In summary, more physiological responses (up-states) would be composed by sequences of cortical stimulus expected to trigger voltage transitions that last over a second. Therefore, up-states represent the product of polysynaptic convergence and are expected to contain all these components. Because up-states represent the convergence that allows the synchronization of SPNs [35,37], then, synchronization of SPNs represents the interaction among cortical and striatal assemblies. Prolonged NMDA- and KA-receptors contributions may allow the time to cross-talk with modulatory signaling purveyed by G-protein coupled receptors. Further work is needed to see whether metabotropic synaptic components are a part of these responses as well as their similarities and differences between dSPNs and iSPNs.

## Methods

All experiments were carried out in accordance with the National Institutes of Health Guide for Care and Use of Laboratory Animals and were approved by the Institutional Animal Care Committee of the Universidad Nacional Autónoma de México. D_1_ and D_2_ dopamine receptor-eGFP BAC transgenic mice, between postnatal days 30–60 (PD30-60; FVB background, developed by the GENSAT project) were used. Adult Wistar rats, wild mice, and non-fluorescent cells of BAC-mice were also recorded to detect possible inconsistencies due to transgenes expression [77]. In all the present cases we obtained consistent and similar results. The number of animals employed in the experimental samples was near the minimal possible to attain robust reproducible results and/or statistical significance. Animals were anesthetized with ketamine/xylazine. Their brains were quickly removed and placed into ice cold (4°C) bath saline containing (in mM): 126 NaCl, 3 KCl, 25 NaHCO_3_, 1 MgCl_2_, 2 CaCl_2_, 11 glucose, 300 mOsm/L, pH = 7.4 with 95% O_2_ and 5% CO_2_. Hemispheres were separated at this stage. Para sagittal (or horizontal in about 20% of cases) neostriatal slices (250–300 μm thick) were cut using a vibratome and stored in oxygenated bath saline at room temperature for at least 1 h before recording.

Intracellular recordings were carried out using sharp microelectrodes (80–120 MΩ) filled with 1% biocytin and 3M potassium acetate fabricated from borosilicate-glass (FHC) and pulled on a Flaming-Brown puller (P-97; Sutter Instruments). Recordings were obtained with a high input impedance electrometer (Neurodata, New York, NY, USA) with an active bridge circuit. Because responses from either dSPNs or iSPNs did not differ as a function of site of the cortex stimulated or slice orientation (parasagittal or horizontal; see Results), most recordings (80%) were performed in parasagittal slices. Slices were submerged in the bath solution and superfused with the same saline at 2 ml/min (34–36°C). Cell membrane potential was ca. -85 mV and input resistance obtained near the resting membrane potential was 50–100 MΏ. Recordings were digitized and stored with the aid of software designed in the laboratory in the Lab View environment (National Ins., Austin, TX, USA). Drugs were dissolved in the bath saline from stock solutions made daily. Some recordings, were carried out using whole-cell patch pipettes filled with (in mM): 115 KH_2_PO_4_, 2 MgCl_2_, 10 HEPES, 0.5 EGTA, 0.2 Na_2_ATP, 0.2 Na_3_GTP and 1% biocytin. Recordings using this internal solution and patch pipettes did not apparently differ from those using sharp electrodes and corresponding solution. After recording, neurons were injected with biocytin as previously described for their identification [25].

Recordings were carried out in the dorsal striatum. Stimulation was performed with concentric bipolar electrodes (tip = 50 μm) to stimulate locally and avoid charge diffusion between cortex and striatum or vice versa. In our experience non-concentric wide open bipolar electrodes frequently produce charge diffusion. The distance between recording and stimulating electrode was around 1 mm for both cortical and intrastriatal stimulations. In many occasions, stimulating electrodes located in the cortex or the striatum were a few microns (500–1000 μm) away of each other and in many cases both sites were stimulated in both locations with the same electrode. In case of current diffusion we may have seen prolonged responses while recording in the striatum. Synaptic responses were evoked by a single square pulse of 0.1 ms. Stimulation was delivered with a stimulator (S-8800; Grass, West Warwick, RI) using an isolation unit. The cell membrane potential was held at −80 mV. A series of current pulses of increasing intensities were used to elicit suprathreshold responses, with or without the firing of repetitive action potentials. The same series of stimulus intensities were used for both intracortical or intrastriatal stimulus. Response magnitudes were measured as areas under the synaptic responses by numerical integration [24]. Responses obtained with suprathreshold stimulus strength (2X threshold) are compared. Statistical values in histograms and text are presented as mean ± SEM. Digital subtraction was used to obtain time courses and the components sensitive to different glutamate receptor antagonists. Normality tests allowed the comparison of paired samples with two tailed Student´s *t* tests. ANOVA and a post hoc Bonferroni test were used when the sample was subject to more than one comparison. Statistical significance was fixed at P < 0.05. In most cases, we used a standard statistical table and report the nearest significant larger value for the corresponding degrees of freedom, so that symbols in the histograms were made homogeneous through different figures.

After recordings, neurons were injected with biocytin. eGFP-positive visualization was observed on a confocal microscope as previously described [25]. Current-clamp data were obtained to observe the most physiological response. A voltage-clamp recording is shown (inset in Figure 1) evoked with low intensity strength to illustrate the barrage of synaptic inputs lasting hundreds of milliseconds after the first monosynaptic response. However, voltage-clamp responses at higher stimulus strengths involve escape currents due to the absence of space-clamp in complex dendritic arbors and the partial filtering introduced by point voltage-clamp that makes physiologically complex suprathreshold responses non-interpretable. eGFP-positive and negative neurons from D_1_ and D_2_ eGFP animals are compared. Dynamic voltage-clamp is out of the scope of the present report.

The following drugs: the *N*-methyl-*D*-aspartate (NMDA) receptor antagonist 5-Phosphono-DL-norvaline DL-2-Amino-5-phosphonovaleric acid (AP-5 or APV), the α-amino-3-hydroxy-5-methyl-isoxazole-4-propionate AMPA and Kainate receptor antagonist 6-Cyano-7-nitroquinoxaline-2,3-dione disodium salt hydrate (CNQX) and bicuculline were obtained from Sigma-RBI. The AMPA receptor antagonist 4-(8-Methyl-9*H*-1,3-dioxolo[4,5-*h*][2,3] benzodiazepin-5-yl)-benzenamine dihydrochloride (GYKI 52466) and the kainate receptor antagonist (*S*)-1-(2-Amino-2-carboxyethyl)-3-(2-carboxy-5-phenylthiophene-3-yl-methyl)-5-methylpyrimidine-2,4-dione (ACET) were obtained from TOCRIS.

## Competing interests

The authors declare that they have no competing interests.

## Authors’ contributions

BJV-C, MAA-G, MBP-R and EF-B did electrophysiological experiments; DT did imaging and immunocytochemistry experiments; RD-C purveyed with ideas, critical discussion and analysis; EG and JB conceived and directed the project, and wrote the manuscript. All authors read and approved the final manuscript.
